# Analyzing the Material Basis of Anti-RSV Efficacy of Lonicerae japonicae Flos Based on the PK-PD Model

**DOI:** 10.3390/molecules28186437

**Published:** 2023-09-05

**Authors:** Yuting Liang, Mingjun Liu, Yanghai Wang, Lu Liu, Yan Gao

**Affiliations:** 1 Institute of Pharmaceutical Research, Shandong University of Traditional Chinese Medicine, Jinan 250355, China; ytliang@cdutcm.edu.cn (Y.L.); 18615693323@163.com (M.L.); 2020110277@sdutcm.edu.cn (Y.W.); lucy1996@126.com (L.L.); 2 High Level Traditional Chinese Medicine Key Disciplines of the State Administration of Traditional Chinese Medicine: Pharmaceutics of Traditional Chinese Medicine, Jinan 250355, China; 3 Collaborative Innovation Center for Ecological Protection and High Quality Development of Characteristic Traditional Chinese Medicine in the Yellow River Basin, Jinan 250355, China

**Keywords:** Lonicerae japonicae Flos, respiratory syncytial virus, effective ingredient, UPLC-MS/MS, PK-PD model

## Abstract

Lonicerae japonicae Flos (LJF) possesses a good anti-respiratory syncytial virus (RSV) effect. However, the material basis of LJF in treating RSV is still unclear. In this study, a sensitive and accurate quantitative method based on UHPLC-QQQ MS was established and validated for the simultaneous determination of the 15 ingredients from LJF in RSV-infected mice plasma. Multiple reaction monitoring was performed for quantification of the standards and of the internal standard in plasma. All the calibration curves show good linear regression within the linear range (r^2^ > 0.9918). The method validation results, including specificity, linearity, accuracy, precision, extraction recovery, matrix effect, and stability of 15 ingredients, are all within the current acceptance criteria. This established method was successfully applied to the pharmacokinetic study of 15 compounds from LJF. Furthermore, the repair rate of lung index and the improvement rate of IFN-γ and IL-6 improved after administration of the LJF, indicating that LJF possessed a positive effect on the treatment of RSV infection. Finally, by combining Spearman and Grey relation analysis, isochlorogenic acid B, isochlorogenic acid C, secoxyloganin, chlorogenic acid, and loganic acid are speculated to be the main effective ingredients of LJF in treating RSV. This study lays the foundation for attempts to reveal the mechanisms of the anti-RSV effect of LJF.

## 1. Introduction

Respiratory syncytial virus (RSV) is a negative sense, single-stranded RNA virus that results in bronchiolitis and asthma with a distinct seasonality [1,2]. For immunocompromised patients, such as children and older people, RSV is a leading cause of disease and death and most children will experience at least one RSV infection by the age of 2 years [3,4]. Unfortunately, there are currently no approved vaccines for RSV, and only Palivizumab and Ribavirin have been approved for the treatment of children at high risk of RSV infection. However, side effects and high costs limit their safety and accessibility, making it urgent to develop new anti-RSV infection drugs that are characterized by their effectiveness, their low toxicity, and their low cost [5]. A cytokine storm in the lungs caused by RSV infection leads to severe pneumonia, in which the inflammatory factors interleukin-6 (IL-6) and interferon-γ (IFN-γ) are considered to be the main markers of severe RSV infection [6,7]. Inflammatory cell infiltration further promotes tissue thickening and hypertrophy, leading to an upward trend in the lung index. Therefore, reducing the inflammatory response in the lungs is an important strategy for suppressing RSV infection.

*Lonicera japonica* Thunb., a well-known medicinal plant, is widely cultivated and used in East Asian countries and regions. The dried flower buds named Lonicerae japonicae flos (LJF), are used as the main medicinal part and possess a variety of pharmacological effects, including effects that are anti-inflammatory, anti-viral and immunomodulatory [8]. A variety of biologically active metabolites have been identified from LJF, such as flavonoids, phenolic acids, saponins, polysaccharides, etc. [9]. LJF extract has been shown to have anti-RSV effects [10]. With further research, an increasing number of bioactive components derived from LJF have been found to be effective in inhibiting the development of RSV. Chlorogenic acid could intervene in RSV infection by regulating the overexpression of TLR3, TBK1, IRF3, and IFN-β, key factors of the TLR3 signal pathway [11]. The 80% alcohol precipitated polysaccharide exhibited the strongest inhibitory activity on RSV in vitro [12]. However, the effective ingredient and the pharmacokinetic characteristics of LJF for the treatment of RSV infection are not fully understood.

Pharmacokinetic (PK) analysis not only contributes to the elucidation of the effects of the target drugs in the body but also to the prediction of the metabolic pattern of drugs. Although the positive effects of the metabolites of biologically active chemical components in the body cannot be ignored, chemical components derived from herbal medicines are usually regarded as potential active ingredients. Therefore, mapping the PK profiles of the prototypical blood-entering components of LJF is an important way to reveal their medicinal effects. However, the concentration of selected target compounds is too low to be detected and there is serious matrix interference, which is the most significant factor limiting the analysis of PK profiles for medicinal plant extracts. Ultra-high performance liquid chromatography-tandem triple quadrupole mass spectrometry (UHPLC-QQQ MS) is becoming the main tool for analyzing PK profiles of medicinal plants because of its ability to achieve rapid and efficient separation of compounds to be tested with different polarities and its high sensitivity when detecting trace compounds [13].

Spearman’s correlation coefficient [14] is independent of the distribution of the variables and the sample size, and is suitable for the study of two variables whose observations are pairs of grades, or the grades obtained by transforming the observations of continuous variables. Grey relation analysis (GRA) [15] is a multi-factor statistical analysis method by which to study the degree of correlation between different objects and factors, and the value of the degree of correlation represents the correlation between the comparison factor and the reference factor. GRA has the advantages of low data requirements, the lack of any requirement to conform to the normal distribution, and a simple principle, and is suitable for the correlation analysis of factors in small samples with unclear connotations, incomplete information, and fewer data. These correlation analysis methods can effectively combine changes in the concentration of prototype components and changes in pharmacodynamic indicators to elucidate the pharmacodynamic material basis of the research object.

In this study, a sensitive and accurate UHPLC-QQQ MS method was developed to determine 15 compounds from LJF in the plasma of RSV-infected mice and was applied to the pharmacokinetic study of orally administered LJF. Spearman’s correlation analysis combined with GRA method was used to analyze the correlation and association of prototypical components with pharmacodynamic indexes in LJF in order to excavate the chemical components that play key roles in the treatment of RSV infection. These analyses will provide new insights and references to unravel the material basis and mechanisms of LJF efficacy in the treatment of RSV infection.

## 2. Results

### 2.1. Selection of 15 Compounds for Pharmacokinetic Study

In our previous study, 39 compounds were identified in LJF based on comprehensive chemical profiling using UPLC-Q-Exactive-Orbitrap-MS (QE) [16]. However, due to diverse bioavailability characteristics of different compounds, not all of these were detected (directly absorbed) in RSV infected mice plasma samples. Therefore, RSV-infected plasma samples collected at different times after oral LJF administration were rapidly analyzed based on the previously established QE method. Fifteen prototype components were successfully identified and selected for pharmacokinetic studies and detailed information is presented in Table 1 and Figure S1.

### 2.2. Optimization of Mass Spectrometry Conditions

**Figure 1:**
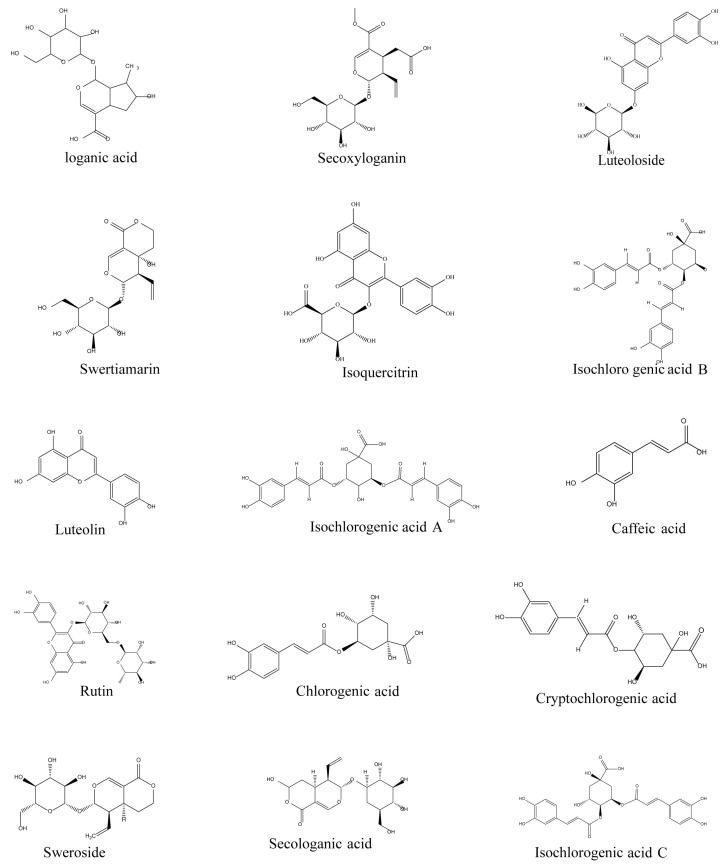
Chemical structures of the 15 compounds from LJF.

**Figure 2:**
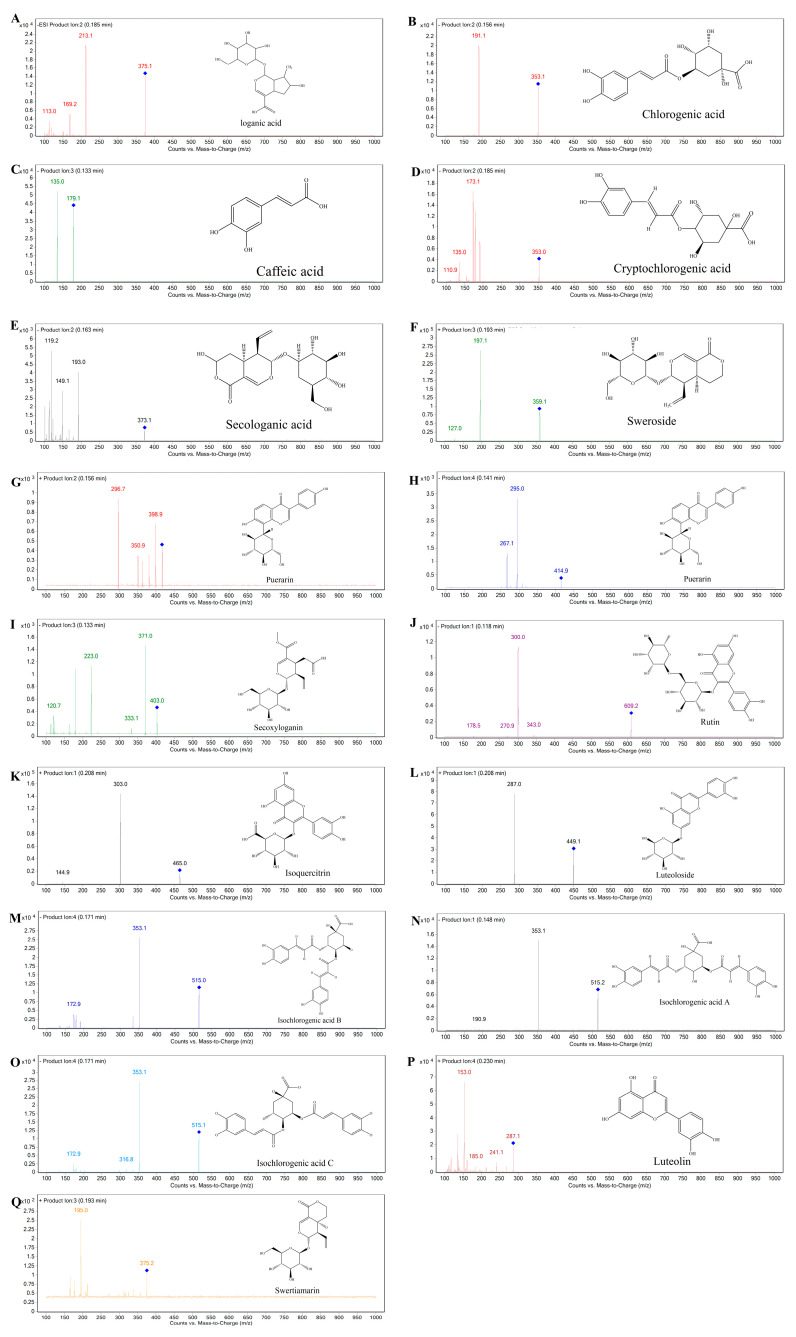
Product ion mass spectra of the 15 compounds from LJF. (**A**) Loganic acid, (**B**) Chlorogenic acid, (**C**) Caffeic acid, (**D**) Cryptochlorogenic acid, (**E**) Secologanic acid, (**F**) Sweroside, (**G**) Puerarin (positive ion mode), (**H**) Puerarin (negative ion mode), (**I**) Secoxyloganin, (**J**) Rutin, (**K**) Isoquercitrin, (**L**) Luteoloside, (**M**) Isochlorogenic acid B, (**N**) Isochlorogenic acid A, (**O**) Isochlorogenic acid C, (**P**) Luteolin, (**Q**) Swertiamarin.

The results show that secologanic acid, cryptochlorogenic acid, isochlorogenic acid B, isochlorogenic acid C, secoxyloganin, caffeic acid, chlorogenic acid, loganic acid, isochlorogenic acid A, rutin, and puerarin (internal standard (IS)) were more sensitive in the negative ion mode, while sweroside, Luteoloside, swertimarin, luteolin, and isoquercitroside were more suitable for detection in the positive ion mode. The multiple reaction monitoring (MRM) mode was selected for the quantitative analysis of 15 analytes due to its suitability for the analysis of compounds with low ionic strength and the possibility of switching the source polarity between positive and negative modes. The chemical structures, ion mass spectra and optimized parameters of the 15 compounds are summarized in Figure 1, Figure 2 and Table 2 respectively.

### 2.3. Method Validation

#### 2.3.1. Specificity

The chromatograms of mice plasma samples collected at 0.5 h after oral administration, blank plasma, and plasma containing standard working solution are portrayed in Figure S2. After processing the plasma, the sample was analyzed as per the above-described method. There was no significant endogenous substance interference at the retention time of the analytes. This established method possessed good specificity.

#### 2.3.2. Linearity and Lower Limit of Quantification (LLOQ)

The calibration curves were plotted by the peak area of the 15 analytes/peak area of IS versus the corresponding concentrations and calculated by weighted (1/x^2^) linear regression. When the calibration curve for each analyte had a value of 10 for the signal-to-noise ratio, the lowest concentration was considered to be the LLOQ. Results show that the relative error (RE) and relative standard deviation (RSD) were specified to be less than 20% at LLOQ. Calibration curves for 15 compounds show a strong linear correlation with R more than 0.9918. The LLOQ distributions of these target components ranged from 1.25–25 ng/mL, suggesting that the developed method possessed good sensitivity. (Table 3).

#### 2.3.3. Accuracy and Precision

QC samples were configured into three concentration gradients. Six QC samples at each concentration were measured on three consecutive days to assess intra-day and inter-day accuracy and precision. The results are shown in Table 4, RSD of the analytes ranged from 1.6% to 10.9% for intra-day precision, 2.4% to 10.7% for inter-day precision and the RE for the accuracy were not exceeded ±10.2% for any analytes. The results show that the method performed satisfactorily to assure a determination of 15 analytes in RSV-infected mice plasma.

#### 2.3.4. Matrix Effects and Extraction Recovery

The matrix effect was evaluated by calculating the ratio of the peak area of a blank plasma sample containing the standard to the peak area of the standard working solution. The extract recoveries were defined as the ratio of the peak area of the individual compounds of the QC sample species to the peak area of the blank plasma containing the different standards. The RSD of matrix effect and extraction recovery of the 15 analytes were all less than 15% (Table 5), indicating that the method was suitable for different matrices, and displayed stable extract recoveries.

#### 2.3.5. Stability

As shown in Table S1, all analytes presented acceptable short-term stability, 24 h stability, long-term stability and freeze–thaw stability, with RSD values less than 14.10%. These results further confirm the stability of the analysis process and storage, providing assurance of the accuracy of the results.

### 2.4. Pharmacokinetics Study

**Figure 3:**
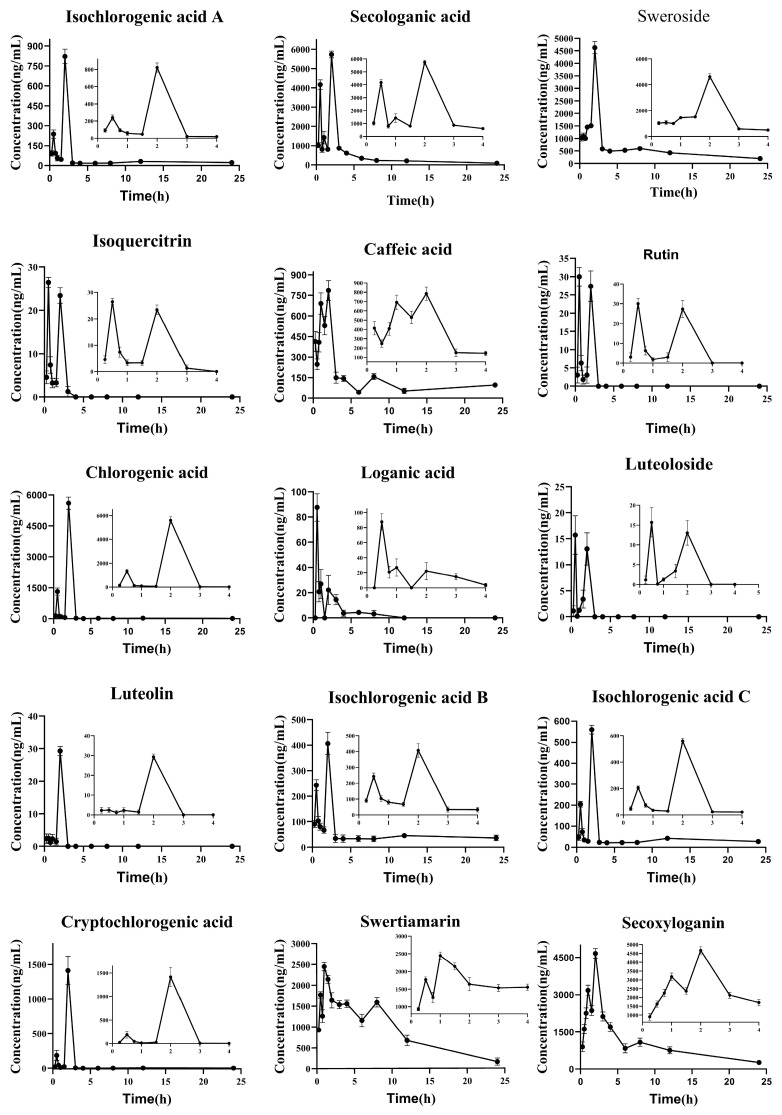
The concentration–time curve of 15 compounds form LJF.

Figure 3 and Table 6 present the drug concentration-time curve and pharmacokinetic data of analytes, respectively. The results indicate that, after oral administration of 12.0 g/kg LJF extract, the pharmacokinetic characteristics of the 15 compounds in mice plasma present significant variability. The concentration–time curve of the isochlorogenic acid A, secologanic acid, isoquercitrin, caffeic acid, rutin, chlorogenic acid, luteoloside, isochlorogenic acid B, isochlorogenic acid C, cryptochlorogenic acid, swertiamarin, and secoxyloganin all displayed double or multiple peaks, which might be caused by impaired enterohepatic circulation, gastrointestinal circulation and gastric emptying [17]. Concentrations of luteoloside and rutin in plasma reached the first peak at 30 min, followed by rapid elimination after reaching peak concentrations again at 2 h, which may be related to the hepatic–intestinal circulation and the greater polarity. In addition, the AUC_0–t_, MRT_0–t_, and T_1/2_ of luteolin were greater than those of luteoloside and their contents were greater than that of lignans, which was hypothesized to be caused by the conversion of lignans to glycosides in the gut and then absorbed into the blood [18]. For the pharmacokinetic parameters of the phenolic acid components, the AUC_0–t_ and C_max_ of caffeic acid were 2.92 × 10^3^ ± 100, 779 ± 19.2, respectively, which were greater than the values of isochlorogenic acid B, isochlorogenic acid A, and isochlorogenic acid C. This might be attributed to the fact that the phenolic acids (chlorogenic, neo-chlorogenic, isochlorogenic, isochlorogenic B, isochlorogenic A, isochlorogenic C) are partially metabolized in the intestine and absorbed as caffeic acid into the blood [19]. The MRT_0–t_ and T_1/2_ of chlorogenic acid B, isochlorogenic acid A, and isochlorogenic acid C were greater than those of chlorogenic acid and cryptochlorogenic acid, suggesting that the elimination of isochlorogenic acid in vivo was much lower than that of chlorogenic acid. This could be due to the high plasma protein binding of isochlorogenic acid, resulting in its slow elimination [20]. The values of C_max_, T_1/2_ and AUC_0–t_ for sweroside (4.63 × 10^3^ ± 243, 10.1 ± 2.25 and 1.42 × 10^4^ ± 729, respectively) were higher than those after a single oral dose of sweroside standard, which might be attributed to the fact that other components in LJF promote the absorption of sweroside [21].

### 2.5. Pharmacodynamic Experiment

**Figure 4:**
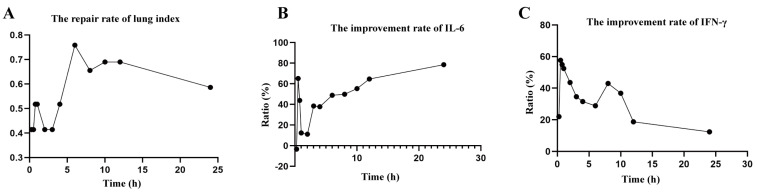
Pharmacodynamic indexes–time curves. (**A**) The repair rate of the lung index–time curve. (**B**) The improvement rate of the IL-6–time curve. (**C**) The improvement rate of the IFN-γ–time curve.

Figure 4 and Table 7 illustrate three pharmacodynamic indexes, the repair rate of lung index, the improvement rate of IFN-γ and the improvement rate of IL-6, which each improved after administration of the LJF and were significantly different from the model group, suggesting that the LJF extract could effectively inhibit lung inflammation in RSV-infected mice. The results show that the lung index and IFN-γ content of RSV-infected mice decreased under 12 different time points after administration, with insignificant differences between groups. At 0.5 h, 0.75 h, 2 h, 3 h, 4 h, 6 h, 8 h, 12 h, and 24 h, the content of IL-6 factor in the lung tissue of mice was significantly reduced. The repair rate of lung index and the improvement rates of IFN-γ and IL-6 peaked at 0.5 h after administration with LJF, which was basically consistent with the time of the first peak of the 15 chemical components. The improvement rates of the three pharmacodynamics reduced gradually over time. Notably, the improvement rates of IFN-γ and IL-6 and the repair rate of lung index peaked again at 3–6 h after administration, which was later than the time of the second peak of drug concentration (2 h after administration), suggesting that there was a phenomenon of delayed pharmacodynamic effect.

### 2.6. Correlation Analysis

#### 2.6.1. Comprehensive Weight Score of Pharmacodynamic Indexes

The entropy value can be used to evaluate the dispersion degree of a given index. A smaller information entropy value indicates a greater dispersion degree of the index, implying that the index has a greater impact on the comprehensive evaluation. The lung index and the efficacy indices (E%) of IL-6 and IFN-γ were scored using the entropy method, and the results are presented in Table S2.

#### 2.6.2. Spearman Correlation Analysis

The results of the Spearman correlation analysis are shown in Table 8. The analysis revealed that the correlation coefficients between the content of five compounds (isochlorogenic acid B, isochlorogenic acid C, secoxyloganin, chlorogenic acid, and loganic acid) and the composite index of efficacy (S) were positively correlated. Conversely, the correlation coefficients between the content of nine compounds, including secologanic acid, sweroside, cryptochlorogenic acid, luteoloside, swertiamarin, luteolin, isoquercitrin, isochlorogenic acid A, and rutin, and the composite index of efficacy (S) were negatively correlated. The *p*-value for each group was greater than 0.05, which was not a significant difference.

#### 2.6.3. Grey Relation Analysis

The results of the GRA indicate that 14 analytes had a significant correlation with S (correlation coefficient > 0.9), which significantly affected the comprehensive efficacy index. In addition, isochlorogenic acid A had a relatively significant effect on the comprehensive efficacy index, with a correlation coefficient between 0.8 and 0.9 with S (Table 9). Therefore, it could be concluded that the 15 components of LJF have a significant impact on the comprehensive efficacy index.

## 3. Discussion

The abundant compounds in LJF have been extensively investigated, and many of them, such as phenolic acids, flavonoids, and cyclic enol ether terpene glycosides are regarded as important chemical markers for evaluating the quality of LJF [22,23]. Several studies have demonstrated the antiviral activity of LJF, including anti-RSV [16], anti-hepatitis B virus [24], and anti-herpesvirus effects [25]. Pharmacokinetic studies are essential for determining the dosage, interval and pharmacological substance basis of drug administration. However, the current pharmacological studies on LJF focus on the pharmacological activities and mechanisms, and less attention has been paid to its pharmacokinetic process in vivo. With the development of detection and analysis techniques, LC-MS has been widely used in the quantitative analysis and pharmacokinetic studies of bioactive components in medicinal plants. The LC-MS method has the advantages of high sensitivity, good selectivity, high resolution, high specificity, and its ability to simultaneously detect multiple ions. Due to the complex chemical properties, the active ingredients in medicinal plant extracts must be transported to the target site using blood as a carrier before they can be effective, so the identification of chemical components in blood is a key strategy to tap the material basis of medicinal efficacy. In this study, the time-varying profiles of the concentrations of 15 blood-entry compounds from LJF were quantified by the UPLC-QQQ-MS method in order to obtain the relevant pharmacokinetic parameters.

The UPLC-QQQ-MS method established in this study successfully quantified 15 effective components with strong specificity in the plasma of RSV-infected mice after oral administration of LJF. The methodological investigation results demonstrate good linearity (r^2^ > 0.990) for all components. Intra-day/inter-day precision, intra-day/inter-day accuracy, repeatability, stability, extraction recovery, and matrix effect were all in accordance with the requirements for the quantitative analysis of biological samples.

LJF extract has been shown to not only reduce levels of IL-6 and TNF-α in order to reduce the inflammatory response but also to combat viral importation and replication by attenuating SARS-CoV-2 M^pro^ activity [26]. Furthermore, LJF is regarded as a core anti-influenza herb, and its key chemical components against the influenza virus have been identified as neochlorogenic acid, chlorogenic acid, and cryptochlorogenic acid [27]. The repair rate of the lung index and the improvement rate of IL-6 and IFN-γ were chosen as the evaluation indexes of drug efficacy. By plotting the efficacy–time curve, we were able to precisely understand the changing pattern of efficacy with time after the administration of LJF. Usually, the drug is absorbed within 0–2 h of entry into the body, and the drug concentration in the blood continues to rise. The concentrations of 15 compounds peaked for the first time in the time period of 0.25–1 h after administration of the LJF, while the repair rate of lung index and the improvement rates of IFN-γ and IL-6 all peaked at 0.5 h. The improvement rates of the three pharmacodynamics again induced progressive declines over time. However, the improvement rate of IFN-γ and the repair rate of lung index peaked again in the 3–6 h time period after administration with LJF. Taken together, we hypothesized that this phenomenon might be due to the way in which the 15 chemical constituents in the LJF extract were first absorbed and metabolized in the body, then excreted by the bile into the hepatic and intestinal circulation, so they were re-absorbed to exert their medicinal effects. This phenomenon was consistent with the occurrence of a double peak in the concentration–time curve, but the second peak in the pharmacological effect occurred later than the second peak in the drug concentration (2 h after administration), suggesting that the pharmacological effect lagged behind the concentration of the drug in plasma.

Spearman correlation analysis and the GRA are appropriate for correlational analysis of various factors in small samples [28,29]. These methods can help determine the contribution of components to efficacy by analyzing the correlation between components and efficacy. As such, Spearman and GRA were selected to analyze the concentration changes and pharmacodynamic changes of the prototype blood components to clarify the pharmacodynamic material basis of LJF against RSV. The results of Spearman correlation analysis show that the correlation coefficients between isochlorogenic acid B, isochlorogenic acid C, secoxyloganin, chlorogenic acid, and loganic acid were positive. Furthermore, the results of the GRA demonstrate that the correlation coefficients between 14 components and S were greater than 0.9. Finally, based on the positive Spearman correlation coefficient between the prototype components, S and the GRA correlation degree being greater than 0.9, the effective material basis of LJF against RSV was speculated as comprising isochlorogenic acid B, isochlorogenic acid C, secoxyloganin, chlorogenic acid, and loganic acid.

Isochlorogenic acid B and isochlorogenic acid C were proved to possess specific anti-RSV effects, which were achieved by inhibiting virus–cell fusion in the early stage and cell–cell fusion at the end of the RSV replication cycle [30]. Chlorogenic acid has a broad spectrum of antiviral activity and could inhibit RSV infection by modulating the expression of TLR3, TBK1, and IRF3 in the TLR3 signaling pathway and decreasing the level of IFN-β [11]. Secoxyloganin plays an important role in the antiviral activity of LJF and may be related to the synergistic effect of multiple compounds [27]. This was in agreement with our results. In addition, loganic acid could effectively prevent viruses from entering cells and possesses significant antiviral activity [31]. These reports strongly support the results of our analysis, which in turn demonstrates the accuracy and reliability of the method of mining the effective material basis by correlation analysis.

## 4. Materials and Methods

### 4.1. Materials and Chemicals

Lonicerae japonicae Flos was purchased from the traditional Chinese medicine trade market (Anhui, China). Puerarin, chlorogenic acid, luteoloside and the internal standard (IS) crinoline were obtained from National Institutes for Food and Drug Control (Beijing, China). Caffeic acid, isochlorogenic acid B, isochlorogenic acid C, and cryptochlorogenic acid were acquired from Chengdu Biopurify Phytochemicals Ltd. (Chengdu, China). Loganic acid, swertiamarin, sweroside, secoxyloganin, and isoquercitrin were acquired from Chengdu Push Bio-technology Co., Ltd. (Chengdu, China). Rutin, secologanic acid, and luteolin were acquired from Shanghai Standard Technology Co., Ltd. (Shanghai, China). Isochlorogenic acid A was acquired from Chengdu Herbpurify Co., Ltd. (Chengdu, China). RSV was provided by Shandong Academy of Medical Sciences (Jinan, China). All standards had a purity of ≥98%.

The acetonitrile, methanol and formic acid were all HPLC grades and purchased from Fisher Scientific (Fair Lawn, NJ, USA). The Elga purification system refined the ultra-pure water for the HPLC-MS/MS analysis and dilutions (Purelab Elga, Britain). Other reagents and chemicals were analytical grades.

### 4.2. Animals

Male BALB/c mice (specific pathogen-free grade, 10–12 g) were obtained from Beijing Vital River Laboratory Animal Technology Co., Ltd. (Beijing, China) (license number: SCXK (jing) 2019-0006). The Ethics Committee on Laboratory Animal Management of Shandong University of Traditional Chinese Medicine approved animal experiment protocols.

### 4.3. Pharmacokinetic Study

#### 4.3.1. Preparation of Sample Solutions

The LJF (12.0 g) were pulverized and soaked in pure water at a solid: liquid ratio of 1:200 and followed by extraction for 30 min. Then, the LJF extract was obtained by filtration, centrifugation (5000 rpm, 10 min) and concentration. Finally, the remains were freeze-dried and stored at −20 °C for subsequent pharmacokinetic assays.

#### 4.3.2. Establishment of Animal Model and Drug Treatment

**Figure 5:**
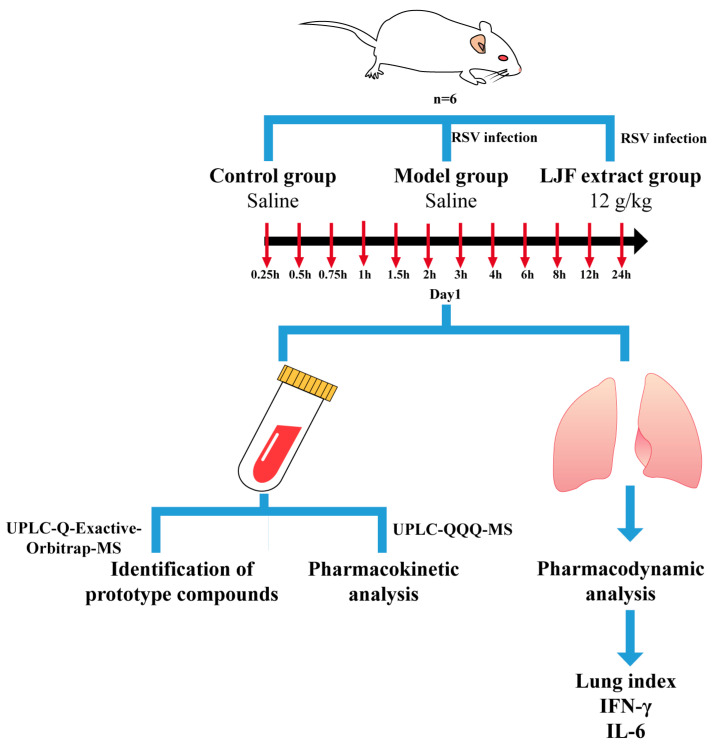
Flowchart of animal experiment.

All mice fasted for 1 night after being acclimatized for 7 days (no water restriction) and were randomly separated into 3 groups with 6 mice in each group. One group of healthy mice was used as the normal control group and administered physiological saline; the model group was established by intranasal infusion of RSV (TCID_50_ = 10^−6^, 35 μL/mouse) and administered physiological saline. Members of the drug group were orally administered with LJF extract at the dose of 12 g/kg after infecting RSV. At 0.25 h, 0.5 h, 0.75 h, 1 h, 1.5 h, 2 h, 3 h,4 h, 6 h, 8 h, 10 h, 12 h and 24 h after administration blood samples (approximately 500 μL) were collected into anticoagulant tubes. The blood samples were centrifuged at 3500 rpm, 4 °C for 10 min, and the plasma (upper layer) was separated and stored at −80 °C till analysis. At the same time, the lung tissue was removed, weighed and placed in the EP tube together with five times the weight of the lung tissue in PBS solution containing 1% PMSF. Subsequently, the tissue samples were homogenized using the tissue grinder, and centrifugation to obtain a supernatant for further examination. The flow of the experiment is shown in Figure 5.

#### 4.3.3. Preparation of Standard Solutions and Quality Control (QC) Samples

Each of the 15 analytes and IS were dissolved in methanol and subsequently mixed appropriately to form a series of standard working solutions. The calibration standard solutions were prepared by mixing appropriate aliquots of the working solution with blank plasma, both containing puerarin (final concentration 2.480 µg/mL). The preparation of QC samples (in low, middle and high concentration levels) was the same as above with blank plasma.

#### 4.3.4. Preparation of Plasma Samples

An amount of 50 μL IS and 2 mL acetonitrile were mixed into 200 μL plasma, after which the mixture was centrifuged (12,000 rpm, 4 °C, 10 min) and dried with nitrogen. The residue was dissolved in 70% acetonitrile containing 1% formic acid, centrifuged and subsequently the supernatant (2 μL) was injected into the UHPLC-QQQ-MS system.

#### 4.3.5. Instruments and Analytical Conditions

Agilent 1260 series (Santa Clara, CA, USA) chromatography system was equipped with a solvent degasser, G1311B quaternary pump and G1329B automated injector. The elution was performed on a Halo^®^ C_18_ column (2.1 × 100 mm, 2.7 μm) at 30 °C, and the mobile phase consisted of 0.05% formic acid in water for solvent A and 0.05% formic acid in acetonitrile for solvent B at a flow rate of 0.3 mL/min. The gradient elution was as follows: 0–30 min at 5–25% B, 30–35 min at 25–55% B, 35–45 min at 55–70% B, 45–55 min at 70–100% B, and 5 μL for analysis.

The MS/MS analysis was performed by Agilent 6470 Triple Quadrupole LC/MS (Santa Clara, CA, USA) with an ESI source. MRM was selected for the detection of the standards and internal standard with a dwell time of 2 ms. The mass spectrometry conditions were optimized as follows: Nebulizer pressure: 50 psi; capillary: positive: 4000 V, negative: 3500 V; cell acceleration voltage: 5 V; gas temp: 350 °C and flow rate at 10 L/min. The acquired data were processed and analyzed by Mass Hunter Workstation quantitative analysis.

Fifteen standards and IS were optimized for mass conditions by the positive and negative ion modes of the chromatographic system, respectively. The pharmacokinetics of 15 chemical constituents in RSV-infected mice were assessed using the UPLC-QQQ-MS/MS method.

#### 4.3.6. Method Validation

The specificity, linearity, lower limit of quantification, accuracy, precision, matrix effect, extract recovery, and stability (storage in a room temperature environment for 4 h, placement in an autosampler at 4 °C for 24 h, storage in a −80 °C environment for 30 days, and 3 repeated freeze–thaw treatments) of the UHPLC-QQQ-MS method was verified in line with the Pharmacopoeia of the People’s Republic of China [32].

#### 4.3.7. Data Analysis of the Pharmacokinetic Study

The pharmacokinetic parameters, including maximum concentration (C_max_), time to reach C_max_ (T_max_), the elimination half-life (T_1/2_), mean residence time (MRT), and area under the concentration–time curve (AUC) were calculated using non-compartmental analysis by Phoenix 8.1.0.3530.

### 4.4. Pharmacodynamic Experiment

#### Pharmacodynamic Index–Time Curve Drawing

The lung index and the repair rate of lung index for each group were calculated using the daily weight values and the weight of lung tissue samples. A curve of the repair rate of lung index over time was plotted using the lung index repair rate as the ordinate and time as the abscissa. The lung tissue samples from each group were homogenized in a PBS solution containing 1% PMSF (five times the weight of the lung tissue), and the supernatant was collected after high-speed centrifugation. The content of IL-6 and IFN-γ was determined using the ELISA method per kit instructions, and the improvement rate was calculated. A curve of the improvement rate of IL-6 and IFN-γ in lung tissue over time after administration was plotted using the improvement rate as the ordinate and time as the abscissa.

### 4.5. Correlation Analysis

#### 4.5.1. Comprehensive Weight Score of Efficacy Index

The data were standardized using dimensionless data to unify the dimensions of each index. The characteristic proportion, information entropy, and weight value of each index were calculated, and the comprehensive score S for each sample evaluation index was obtained.

#### 4.5.2. Spearman Correlation Analysis

The data matrix was constructed using GraphPad Prism 8.0 software with the concentration of 15 components quantitatively detected at specific time points (0.25 h, 0.5 h, 0.75 h, 1 h, 1.5 h, 2 h, 3 h, 4 h, 6 h, 8 h, 12 h, and 24 h) after administration as the independent variable and the comprehensive pharmacodynamic index S as the dependent variable. The original data were standardized using Z-score, and the non-parametric test was used to calculate the Spearman correlation coefficient and *p* value of the X and Y data.

#### 4.5.3. Grey Relation Analysis

The original data for each group were transformed into non-dimensional data using the mean transformation method. The comprehensive efficacy index S was used as the characteristic sequence, and the contents of 15 components at different time points were used as the related factor sequence. These were then input into the GRA calculation formula to determine the correlation degree.

## 5. Conclusions

In this study, a proprietary analytical method was developed to accurately quantify 15 components in the plasma of RSV-infected mice after oral administration of LJF. The repair rate of lung index and the improvement rate of IL-6 and IFN-γ indicated that LJF was effective in relieving lung inflammation caused by RSV infection. PK-PD analysis reveals the changing patterns of 15 components from LJF in RSV-infected mice. In addition, correlation analyses facilitated the identification of isochlorogenic acid B, isochlorogenic acid C, secoxyloganin, chlorogenic acid, and loganic acid as the pharmacological substance basis for the anti-RSV effect of LJF. This research strategy is a feasible and accurate method to reveal the pharmacological substance basis of medicinal plants.

## Figures and Tables

**Table 1:** Quick identification of 15 compounds of LJF in RSV infected mice plasma samples.

Compound	Formula	Precursor Ion (*m*/*z*)	t_R_ (min)	Fragment Ions (*m*/*z*)
Secologanic acid	C_16_H_22_O_10_	373.1143 [M − H]^−^	3.99	179.0549, 149.0595, 123.0438, 59.0122
Sweroside	C_16_H_22_O_9_	359.1336 [M + H]^+^	4.72	127.0393, 179.0705, 197.0811, 151.0757, 111.0810
Chlorogenic acid	C_16_H_18_O_9_	353.0883 [M − H]^−^	3.55	191.0553, 135.0439, 173.0445
Cryptochlorogenic acid	C_16_H_18_O_9_	353.0880 [M − H]^−^	3.91	191.0553, 135.0438, 173.0445, 93.0330, 85.0279
Luteoloside	C_21_H_20_O_11_	447.0955 [M − H]^−^	7.9	151.0024, 447.0955
Isochlorogenic acid B	C_25_H_24_O_12_	515.1198 [M − H]^−^	6.13	353.0882, 191.0553, 173.0445, 135.0438, 93.0330
Isochlorogenic acid A	C_25_H_24_O_12_	515.1201 [M − H]^−^	7.37	353.0882, 191.0553, 173.0445, 135.0438
Isochlorogenic acid C	C_25_H_24_O_12_	515.1201 [M − H]^−^	8.1	353.0883, 191.0554, 173.0446, 135.0439
Swertiamarin	C_16_H_22_O_10_	373.1143 [M − H]^−^	3.99	373.1143, 193.0499, 149.0595, 97.0279
Luteolin	C_15_H_10_O_6_	287.0552 [M + H]^+^	10.07	153.0184, 135.0443
Isoquercitrin	C_21_H_20_O_12_	463.0890 [M − H]^−^	6.68	151.0025, 301.0349
Secoxyloganin	C_17_H_24_O_11_	403.1252 [M − H]^−^	5.39	139.0025, 121.0281, 223.0609
Caffeic acid	C_9_H_8_O_4_	179.0340 [M − H]^−^	4.09	135.0439
Rutin	C_27_H_30_O_16_	609.1461 [M − H]^−^	6.43	300.0279

**Table 2:** Optimized MRM parameters of the 15 compounds in LJF and IS.

Components	Precursor Ion (*m*/*z*)	Product Ion (*m*/*z*)	Fragmentor (V)	Collision Energy (V)	Detected Ion
Secologanic acid	373.1	192.9	245	15	[M − H]^−^
Sweroside	359.1	197.1	70	2	[M + H]^+^
Cryptochlorogenic acid	353.1	173.1	230	10	[M − H]^−^
Luteoloside	449.0	287.1	105	10	[M + H]^+^
Isochlorogenic acid B	515.1	353.0	140	18	[M − H]^−^
Swertiamarin	375.0	194.9	70	7	[M + H]^+^
Luteolin	287.0	153.0	185	30	[M + H]^+^
Isoquercitrin	465.1	303.1	95	4	[M + H]^+^
Isochlorogenic acid C	515.1	353.0	140	10	[M − H]^−^
Secoxyloganin	403.0	371.3	230	9	[M − H]^−^
Caffeic acid	179.1	135.0	105	9	[M − H]^−^
Chlorogenic acid	353.2	191.1	210	8	[M − H]^−^
Loganic acid	375.1	213.1	270	15	[M − H]^−^
Isochlorogenic acid A	515.1	353.0	140	8	[M − H]^−^
Rutin	609.1	300.0	230	32	[M − H]^−^
Puerarin	417.3	296.9	140	25	[M + H]^+^
Puerarin	415.1	295.1	195	20	[M − H]^−^

**Table 3:** Calibration curve, R^2^, linear range and LLOQs of the 15 compounds from LJF.

Components	Calibration Curve	(Correlation Coefficient) R^2^	Linear Range (ng/mL)	LLOQ (ng/mL)
Secologanic acid	y = 0.0001x − 0.1336	0.9967	40,000.000–50.000	50.000
Sweroside	y = 0.0196x + 4.4734	0.9969	25,000.000–3.125	3.125
Cryptochlorogenic acid	y = 0.0007x + 0.0249	0.9982	2.500–5000.000	2.500
Luteoloside	y = 0.0256x + 0.0725	0.9998	1.250–1000.000	1.250
Isochlorogenic acid B	y = 0.0042x − 2.6816	0.996	25.000–25,000.000	25.000
Swertiamarin	y = 0.00008 + 0.0219	0.9939	50.000–20,000.000	50.000
Luteolin	y = 0.0194x + 0.1584	0.9935	1.250–4000.000	1.250
Isoquercitrin	y = 0.0132x − 0.1004	0.9991	2.500–4000.000	2.500
Isochlorogenic acid C	y = 0.0093x − 1.7728	0.9918	12.500–10,000.000	12.500
Secoxyloganin	y = 0.0008x − 0.202	0.9994	5.000–40,000.000	5.000
Caffeic acid	y = 0.0092x − 0.3147	0.9949	50.000–20,000.000	50.000
Chlorogenic acid	y = 0.0019x + 0.0455	0.9994	1.875–7500.000	1.875
Loganic acid	y = 0.0003x + 0.0243	0.9971	2.500–10,000.000	2.500
Isochlorogenic acid A	y = 0.0069x − 2.1725	0.9989	5.000–20,000.000	5.000
Rutin	y = 0.0050x − 0.0593	1.0000	2.500–4000.000	2.500

**Table 4:** Methodology verification results of precision and accuracy.

Components	Concentration (ng/mL)	Inter-Day		Intra-Day	
		Accuracy (RE%)	Precision (RSD%)	Accuracy (RE%)	Precision (RSD%)
Secologanic acid	100.00	−6.10	4.60	−6.30	5.40
	8000.00	−7.30	5.30	−7.80	6.70
	30,000.00	−5.50	6.80	−5.40	6.30
Sweroside	6.25	−7.40	8.10	−7.10	3.40
	5000.00	−6.05	4.90	−8.30	5.90
	18,750.00	−4.30	5.20	−7.90	3.60
Cryptochlorogenic acid	6.25	−5.60	5.40	−2.40	7.80
	2000.00	−6.30	4.70	−5.50	9.10
	5000.00	−4.80	7.20	−4.90	8.20
Luteoloside	6.25	−9.60	7.50	−7.20	4.70
	100.00	−10.20	5.80	−6.80	5.20
	1000.00	−8.90	6.60	−5.70	9.40
Isochlorogenic acid B	500.00	−6.70	5.70	−5.20	7.10
	5000.00	−10.30	2.90	−6.40	8.50
	18,750.00	−2.10	3.80	−7.10	4.30
Swertiamarin	400.00	−8.90	6.80	−2.60	7.80
	4000.00	−7.60	6.40	−9.80	10.20
	15,000.00	−9.70	4.50	−7.30	9.80
Luteolin	6.25	−2.90	3.90	−4.90	2.10
	200.00	−6.30	3.10	−5.60	1.80
	3000.00	−6.70	8.60	−1.90	3.20
Isoquercitrin	50.00	−8.20	6.60	−3.80	10.90
	200.00	−7.30	5.40	−6.20	7.30
	3000.00	−4.80	7.20	−5.30	6.40
Isochlorogenic acid C	200.00	−10.60	2.40	−4.50	2.70
	4000.00	−4.80	4.50	−2.90	3.80
	7500.00	5.20	5.70	−5.80	1.60
Secoxyloganin	10.00	−4.30	6.80	−6.10	5.10
	400.00	−4.80	6.60	−5.50	3.50
	30,000.00	−7.40	8.40	−5.70	6.70
Caffeic acid	400.00	−6.80	10.70	−4.30	8.30
	4000.00	−7.60	5.80	−4.10	6.10
	15,000.00	−9.80	3.40	−2.60	4.90
Chlorogenic acid	3.75	−8.10	8.80	−5.30	6.10
	300.00	−7.60	6.40	−8.10	5.20
	5625.00	−6.40	9.30	−6.90	7.10
Loganic acid	100.00	−5.80	5.10	−5.40	5.90
	2000.00	−7.20	6.70	−6.00	9.20
	7500.00	−3.40	7.50	−3.70	5.60
Isochlorogenic acid A	400.00	−5.60	5.60	−4.20	8.10
	5000.00	−8.10	2.70	−4.30	4.70
	15,000.00	−5.40	5.40	−8.90	6.30
Rutin	5.00	−5.30	3.90	−8.40	5.40
	200.00	−6.10	9.10	−6.50	4.60
	3000.00	−3.70	7.20	−5.70	6.30

**Table 5:** The recovery and matrix effect of the compounds in mice plasma (n = 6).

Components	Concentration	Recovery (%)	Matrix Effect (%)
	(ng/mL)	(Mean ± SD)	(Mean ± SD)
Secologanic acid	100	86.1 ± 5.3	88.6 ± 6.3
	8000	87.3 ± 4.5	92.0 ± 2.8
	30,000	92.5 ± 6.1	86.7 ± 7.4
Sweroside	6.25	87.4 ± 2.9	87.2 ± 5.1
	5000	91.5 ± 5.2	96.1 ± 4.3
	18,750	92.3 ± 6.1	87.9 ± 6.4
Cryptochlorogenic acid	6.25	88.6 ± 7.4	92.4 ± 3.8
	2000	86.3 ± 8.2	91.5 ± 5.7
	5000	92.8 ± 5.8	85.9 ± 6.3
Luteoloside	6.25	89.6 ± 5.2	87.3 ± 7.2
	100	90.2 ± 6.5	96.4 ± 5.7
	1000	88.9 ± 7.1	95.5 ± 7.3
Isochlorogenic acid B	500	86.7 ± 3.6	85.2 ± 6.1
	5000	91.3 ± 8.4	93.5 ± 7.3
	18,750	92.1 ± 5.8	87.1 ± 4.5
Swertiamarin	400	88.9 ± 6.7	92.6 ± 5.3
	4000	87.6 ± 5.2	89.8 ± 5.1
	15,000	89.7 ± 5.4	87.3 ± 6.3
Luteolin	6.25	92.5 ± 6.1	94.2 ± 7.2
	200	88.3 ± 4.9	85.7 ± 5.4
	3000	89.5 ± 5.4	91.3 ± 6.8
Isoquercitrin	50	88.2 ± 6.5	93.8 ± 7.2
	200	87.3 ± 6.4	96.2 ± 3.7
	3000	92.3 ± 5.8	95.3 ± 4.8
Isochlorogenic acid C	200	90.6 ± 7.3	87.5 ± 6.1
	4000	91.8 ± 4.9	92.2 ± 5.2
	7500	92.2 ± 7.2	87.5 ± 3.9
Secoxyloganin	10	91.6 ± 8.4	96.1 ± 4.7
	400	92.8 ± 9.2	85.5 ± 6.4
	30,000	87.4 ± 2.5	95.2 ± 3.8
Caffeic acid	400	86.8 ± 5.3	87.3 ± 6.4
	4000	87.6 ± 6.1	94.1 ± 5.3
	15,000	89.8 ± 7.4	92.2 ± 2.5
Chlorogenic acid	3.75	88.1 ± 5.6	95.3 ± 5.6
	300	87.6 ± 7.3	90.0 ± 4.2
	5625	90.4 ± 8.2	86.9 ± 7.8
Loganic acid	100	93.8 ± 5.8	92.4 ± 5.4
	2000	87.2 ± 5.3	86.0 ± 6.2
	7500	93.0 ± 8.9	93.0 ± 5.1
Isochlorogenic acid A	400	91.6 ± 6.4	86.9 ± 8.3
	5000	88.1 ± 5.6	92.3 ± 7.2
	15,000	87.5 ± 4.8	88.9 ± 5.2
Rutin	5	89.3 ± 5.2	88.4 ± 6.1
	200	93.1 ± 4.3	86.5 ± 6.2
	3000	92.7 ± 7.8	85.7 ± 8.1

**Table 6:** Pharmacokinetic parameters of 15 components in LJF after oral administration in RSV mice ($\bar{x}$ ± SD, n = 6).

Components	AUC_(0–t)_/mg/L*h	AUC_(0–∞)_/mg/L*h	T_1/2_/h	T_max_/h	C_max_/mg/L	MRT_(0–t)_/h
Secologanic acid	1.21 × 10^4^ ± 305	1.35 × 10^4^ ± 293	10.9 ± 2.46	2.00 ± 0.00	5.73 × 10^3^ ± 173	4.88 ± 0.0632
Rutin	31.3 ± 4.34	34.3 ± 3.33	2.03 ± 1.10	0.500 ± 0.00	30.0 ± 2.54	1.51 ± 0.0291
Isochlorogenic acid A	1.07 × 10^3^ ± 36.3	1.45 × 10^3^ ± 145	26.6 ± 4.52	2.00 ± 0.00	822 ± 53.9	4.64 ± 0.289
Loganic acid	84.9 ± 12.8	99.3 ± 18.4	2.33 ± 0.429	0.500 ± 0.00	87.6 ± 10.9	2.29 ± 0.337
Chlorogenic acid	5.19 × 10^3^ ± 290	5.90 × 10^3^ ± 458	26.1 ± 9.42	2.00 ± 0.00	5.60 × 10^3^ ± 299	2.95 ± 0.0749
Caffeic acid	2.92 × 10^3^ ± 100	3.28 × 10^3^ ± 112	7.92 ± 1.10	2.00 ± 0.00	779 ± 19.2	5.92 ± 0.239
Secoxyloganin	2.44 × 10 ± 707	2.77 × 10^4^ ± 1.50 × 10^3^	8.14 ± 1.28	2.00 ± 0.00	4.67 × 10^3^ ± 207	7.19 ± 0.139
Isochlorogenic acid C	926 ± 39.0	1.45 × 10^3^ ± 118	26.9 ± 3.16	2.00 ± 0.00	560 ± 20.7	6.39 ± 0.337
Isoquercitrin	26.5 ± 1.19	39.6 ± 8.47	2.95 ± 1.72	0.500 ± 0.00	26.5 ± 1.16	1.50 ± 0.0366
Luteolin	20.9 ± 5.37	21.3 ± 5.32	1.96 ± 0.746	2.00 ± 0.00	19.6 ± 6.11	2.12 ± 0.149
Swertiamarin	2.15 × 10^4^ ± 1.67 × 10^3^	2.29 × 10^4^ ± 2.56 × 10^3^	5.15 ± 1.52	1.00 ± 0.00	2.45 × 10^3^ ± 101	7.37 ± 0.540
Isochlorogenic acid B	1.10 × 10^3^ ± 22.4	1.74 × 10^3^ ± 97.5	24.4 ± 2.72	2.00 ± 0.00	415 ± 4.69	7.35 ± 0.170
Luteoloside	15.8 ± 0.991	15.9 ± 1.01	0.343 ± 0.0915	0.500 ± 0.00	16.5 ± 1.97	1.50 ± 0.0928
Cryptochlorogenic acid	1.26 × 10^3^ ± 149	1.32 × 10^3^ ± 140	13.5 ± 2.98	2.00 ± 0.00	1.41 × 10^3^ ± 204	2.79 ± 0.225
Sweroside	1.42 × 10^4^ ± 729	1.72 × 10^4^ ± 1.61 × 10^3^	10.1 ± 2.25	2.00 ± 0.00	4.63 × 10^3^ ± 243	7.17 ± 0.274

**Table 7:** Three pharmacodynamic indexes of RSV-infected mice after administration of LJF in each group (n = 6, $\bar{x}$ ± s).

Group	Lung Index (%)	Repair Rate (%)	IL-6 (pg/mL)	Improvement Rate (%)	IFN-γ (pg/mL)	Improvement Rate (%)
C	0.78 ± 0.03		8.917 ± 1.79		20.01 ± 0.98	
M	1.07 ± 0.07 ^##^		20.19 ± 1.65 ^##^		8.36 ± 0.85 ^##^	
0.25 h	0.99 ± 0.04 *	0.27	20.57 ± 1.53	−3.37	10.92 ± 0.77 **	21.95
0.5 h	0.92 ± 0.06 *	0.51	12.85 ± 1.03 **	65.11	15.10 ± 0.83 **	57.63
0.75 h	0.94 ± 0.07 *	0.44	15.25 ± 0.78 **	43.82	14.76 ± 0.92 **	54.88
1 h	0.94 ± 0.08 **	0.44	18.80 ± 1.45	12.33	14.47 ± 0.90 **	52.40
1.5 h	0.95 ± 0.05 **	0.41	18.93 ± 1.51	11.17	13.44 ± 0.86 **	43.56
2 h	0.95 ± 0.04 *	0.41	15.85 ± 1.50 **	38.49	12.38 ± 0.77 **	34.56
3 h	0.92 ± 0.08 *	0.51	15.93 ± 1.08 **	37.78	12.07 ± 0.93 **	31.47
4 h	0.85 ± 0.02 **	0.75	14.68 ± 0.54 **	48.87	11.70 ± 0.95 **	28.81
6 h	0.88 ± 0.07 **	0.65	14.57 ± 0.88 **	49.85	13.38 ± 0.87 **	42.96
8 h	0.87 ± 0.03 **	0.69	13.95 ± 1.13 **	55.35	12.65 ± 0.99 **	36.79
12 h	0.87 ± 0.05 **	0.69	12.90 ± 0.44 **	64.66	10.51 ± 1.09 **	18.69
24 h	0.90 ± 0.07 **	0.58	11.34 ± 1.56 **	78.50	9.80 ± 0.87 *	12.34

Note: Compared with the control group (C), *p* * < 0.05, *p* ** < 0.01. Compared with the model group (M), *p* ^##^ < 0.01.

**Table 8:** The results of the Spearman correlation analysis.

Components	Correlation (S)		
	No.	R	*p*
Secologanic acid	1	−0.0139	0.9739
Sweroside	2	−0.2098	0.5137
Cryptochlorogenic acid	3	−0.0699	0.8346
Luteoloside	4	−0.2378	0.4573
Isochlorogenic acid B	5	0.2098	0.5137
Swertiamarin	6	−0.2308	0.4708
Luteolin	7	−0.1049	0.7493
Isoquercitrin	8	−0.1818	0.5731
Isochlorogenic acid C	9	0.1678	0.6039
Secoxyloganin	10	0.0769	0.8171
Caffeic acid	11	0	0.9999
Chlorogenic acid	12	0.0559	0.8692
Loganic acid	13	0.2491	0.4308
Isochlorogenic acid A	14	0.1538	0.6353
Rutin	15	−0.1088	0.7401

**Table 9:** Results of the Grey relation analysis.

Components	No.	Correlation (S)
		R
Secologanic acid	1	0.9975
Sweroside	2	0.9980
Cryptochlorogenic acid	3	0.9962
Luteoloside	4	0.9961
Isochlorogenic acid B	5	0.9978
Swertiamarin	6	0.9986
Luteolin	7	0.9966
Isoquercitrin	8	0.9972
Isochlorogenic acid C	9	0.9973
Secoxyloganin	10	0.9986
Caffeic acid	11	0.9981
Chlorogenic acid	12	0.9962
Loganic acid	13	0.9977
Isochlorogenic acid A	14	0.8414
Rutin	15	0.9867

## Data Availability

All data generated or analyzed during this study are included in this article.

## Supplementary Materials

The following supporting information can be downloaded at: https://www.mdpi.com/article/10.3390/molecules28186437/s1, Table S1. The stability of the 15 compounds in plasma at different levels (n = 6). Table S2. The comprehensive score of pharmacodynamic index value entropy method. Figure S1. Chromatogram of plasma samples from RSV-infected mice after oral administration of LJF extract at 2 h. (A) Positive Ion Mode. (B) Negative Ion Mode. Figure S2. MRM chromatograms of the 15 compounds and IS. (A) The chromatograms of mice plasma samples at 0.5 h after oral administration. (B) The chromatograms of plasma containing standard working solution. (C). The chromatograms of blank plasma. Note: 1. Loganic acid, 2. Chlorogenic acid, 3. Caffeic acid, 4. Cryptochlorogenic acid, 5. Secologanic acid, 6. Swertiamarin, 7. Sweroside, 8. Puerarin (Positive ion mode), 9. Puerarin (Negative ion mode), 10. Secoxyloganin, 11. Rutin, 12. Isoquercitrin, 13. Luteoloside, 14. Isochlorogenic acid B, 15. Isochlorogenic acid A, 16. Isochlorogenic acid C, 17. Luteolin.

## Sample Availability

Samples of the Lonicerae japonicae Flos are available from the authors.
