# Supplementary material for: Analyzing the Material Basis of Anti-RSV Efficacy of Lonicerae japonicae Flos Based on the PK-PD Model
Source: Molecules. 2023 Sep 5;28(18):6437. doi: 10.3390/molecules28186437 (PMC10537356; doi:10.3390/molecules28186437)
Supplement: Supplementary file 1 [file molecules-28-06437-s001.zip › Supplementary Materials.pdf]

Supplementary Materials

Table S1 The stability of the 15 compounds in plasma (n = 6).

Components	Concentration (ng/mL)	Short term stability		24 h stability		Long term stability		Repeated freezing and thawing stability	
		Mean ± SD %	RSD %	Mean ± SD %	RSD %	Mean ± SD %	RSD %	Mean ± SD %	RSD %
Secologanic acid	100	98.2±5.1	5.2	94.3±4.1	4.3	87.3±5.6	6.4	90.2±4.6	5.1
	8000	97.3±6.3	6.5	96.3±7.3	7.6	89.5±6.2	6.9	92.4±5.1	5.5
	30000	96.4±4.5	4.7	91.4±6.6	7.2	88.3±3.6	4.1	87.2±4.7	5.4
Sweroside	6.25	95.2±6.4	6.7	92.2±3.3	3.6	89.5±5.4	6.0	88.4±4.6	5.2
	5000	98.3±7.0	7.1	95.3±6.6	6.9	90.1±4.8	5.3	89.5±4.6	5.1
	18750	97.4±5.3	5.4	96.4±5.4	5.6	87.4±6.2	7.1	90.8±4.2	4.6
Cryptochlorogenic acid	6.25	96.5±6.6	6.8	91.4±5.7	6.2	89.4±4.5	5.0	92.3±4.3	4.7
	2000	94.2±4.3	4.6	92.8±6.2	6.7	89.8±5.4	6.0	91.5±3.7	4.0
	5000	97.4±5.8	6.0	93.4±4.7	5.0	87.6±3.6	4.1	90.2±6.8	7.5
Luteoloside	6.25	99.5±4.6	4.6	96.4±6.7	7.0	90.7±5.6	6.2	92.6±4.5	4.9
	100	97.3±8.2	8.4	91.3±3.4	3.7	87.3±4.9	5.6	90.2±4.6	5.1
	1000	100.2±2.9	2.9	98.2±3.9	4.0	90.8±8.6	9.5	94.3±5.8	6.2
Isochlorogenic acid B	500	100.3±6.5	6.5	96.3±5.5	5.7	92.4±6.3	6.8	91.5±3.7	4.0
	5000	94.9±4.3	4.5	92.1±3.2	3.5	89.1±5.4	6.1	90.1±5.2	5.7
	18750	95.2±2.1	2.2	90.2±4.1	4.5	88.2±4.1	4.6	91.2±5.4	5.9
Swertiamarin	400	98.2±4.9	5.0	91.4±3.6	3.9	88.5±5.3	6.0	90.1±4.2	4.7
	4000	99.1±6.6	6.7	93.1±5.4	5.8	89.1±4.3	4.8	92.3±2.9	3.1

	15000	98.1±8.7	8.9	90.1±5.4	6.0	87.7±8.1	9.2	92.3±4.1	4.4
	6.25	101.2±3.9	3.9	92.5±5.9	6.4	89.5±3.6	4.02	94.5±4.5	4.8
Luteolin	200	103.8±6.3	6.2	96.8±4.5	4.6	90.5±6.2	6.8	92.4±6.0	6.5
	3000	98.5±6.7	6.8	93.2±4.1	4.4	87.3±6.3	7.2	90.8±2.2	2.4
	50	97.3±8.2	8.4	91.5±2.8	3.0	85.5±3.4	4.0	92.6±4.3	4.6
Isoquercitrin	200	92.1±4.3	4.7	89.1±2.3	2.6	87.3±4.2	4.8	90.8±5.6	6.2
	3000	97.2±4.8	4.9	88.6±4.5	5.1	86.3±5.3	6.1	92.5±5.1	5.5
	200	91.2±1.6	1.8	86.2±2.7	3.1	85.6±4.1	4.8	88.6±4.3	4.9
Isochlorogenic acid C	4000	102.3±4.7	4.6	92.3±3.9	4.2	89.3±5.3	5.9	90.2±4.9	5.4
	7500	101.1±5.2	5.2	91.1±4.5	4.9	88.1±6.2	7.0	92.8±3.1	3.3
	10	99.3±4.3	4.3	93.2±5.2	5.6	89.1±4.8	5.4	92.1±6.8	7.4
Secoxyloganin	400	98.2±4.5	4.6	91.2±3.4	3.7	86.6±6.5	7.5	95.6±5.4	5.6
	30000	96.3±7.4	7.7	91.3±5.3	5.8	88.7±7.4	8.3	89.5±6.3	7.0
	400	98.2±6.8	6.9	93.1±3.8	4.1	87.5±4.6	5.3	90.5±5.8	6.4
Caffeic acid	4000	94.5±6.6	7.0	87.6±2.8	3.2	86.3±4.7	5.4	91.2±4.9	5.4
	15000	94.3±4.8	5.1	88.3±5.6	6.3	87.1±5.1	5.9	90.8±6.6	7.3
	3.75	97.6±7.1	7.3	91.6±5.2	5.7	85.9±6.4	7.5	92.3±6.2	6.7
Chlorogenic acid	300	97.2±5.6	5.8	94.6±6.2	6.6	89.6±7.1	7.9	92.6±4.5	4.9
	5625	96.3±5.4	5.6	92.3±3.5	3.8	88.3±4.6	5.2	90.5±5.5	6.1
	100	95.4±5.8	6.2	90.4±4.8	5.3	89.2±5.2	5.8	92.6±4.4	4.8
Loganic acid	2000	94.3±6.2	6.6	88.5±5.4	6.1	86.6±6.3	7.3	91.2±7.3	8.0
	7500	97.5±3.4	3.5	91.6±4.5	4.9	89.4±5.1	5.7	91.6±8.2	9.0
	400	96.3±5.6	5.8	89.4±4.7	5.3	87.5±5.3	6.1	90.8±5.9	6.5
Isochlorogenic acid A	5000	98.6±2.5	2.5	90.5±5.1	5.6	87.5±6.5	7.4	93.6±4.5	4.8
	15000	99.4±5.4	5.4	87.3±3.7	4.2	86.1±5.1	5.9	94.5±5.8	6.1

	5	97.8±5.3	5.4	89.9±5.3	5.9	87.6±4.5	5.1	95.6±4.8	5.0
Rutin	200	97.5±6.1	6.3	88.6±4.5	5.1	86.9±3.6	4.2	93.2±6.3	6.8
	3000	98.4±3.7	3.8	95.3±5.1	5.4	89.4±6.3	7.0	91.5±4.1	4.5

Table S2. The comprehensive score of pharmacodynamic index value entropy method.

Time (h)	E%			Comprehensive weight score
	Lung index	IL-6	IFN-γ	S
0.25	0.2037	0.1515	4.5951	0.0109
0.5	1.7213	0.5825	1.5736	0.0064
0.75	4.5223	3.1324	3.5757	0.0255
1	4.5672	3.9497	3.8490	0.0290
1.5	1.6378	0.0339	0.0349	0.0029
2	3.8842	3.4983	3.5448	0.0243
3	0.8043	3.2398	3.3743	0.0153
4	1.9507	3.9154	3.7876	0.0210
6	2.0811	2.8981	3.0669	0.0162
8	1.0543	0.9386	1.4969	0.0055
12	4.1837	3.1547	3.3242	0.0237
24	4.3369	3.4858	3.8358	0.0266
